# A new species of *Mallinella* Strand, 1906 (Araneae, Zodariidae) from South China

**DOI:** 10.3897/BDJ.11.e105513

**Published:** 2023-06-20

**Authors:** Zimin Jiang, Yanbin Yao, Yonghong Xiao, Keke Liu

**Affiliations:** 1 Jinggangshan University, Ji'an, China Jinggangshan University Ji'an China; 2 Jinshan College of Fujian Agriculture And Forestry University, Fuzhou, China Jinshan College of Fujian Agriculture And Forestry University Fuzhou China

**Keywords:** Jiangxi Province, spider, taxonomy, zodariid species

## Abstract

**Background:**

Only one zodariid species, *Storenomorphalushanensis* Yu & Chen, 2009 was found from Jiangxi Province. No other *Mallinella* species have been recorded from this Province.

**New information:**

A new species, *Mallinellashahu* **sp. n.** is described from Jiangxi Province, China. Morphological illustrations, living photos and distribution map are given.

## Introduction

The spider family Zodariidae Thorell,1881 is one of the most species-rich families recorded from the world (World Spider Catalog 2023). They usually live in leaf litter, woody debris, under stones or on the forest floor. Currently, there are 1264 species from 90 genera worldwide, with only 55 species belonging to eight genera being recorded from China. Most of them (55 species) are reported from the southern provinces of the country. At present, the genus *Mallinella* Strand, 1906 is the most diverse group in this family. According to the collecting information recorded on World Spider Catalog (2023), all of these *Mallinella* species are found from the southern provinces, such as Guangdong (one species), Guangxi (five species), Hainan (six species), Hunan (six species), Tibet (one species), Yunnan (four species), Guizhou (two species) and Zhejiang (one species). No species have been recorded from other provinces in this large country.

The genus *Mallinella* is characterised by the presence of a single row of ventral spines situated in front of the spinnerets, the palpal tibia with one or three apophyses, the conductor consisting of several parts, the embolic base connected to a tegulum via a thin membrane, the epigyne with a deep anterior median incision and the short and diverging copulatory ducts (Dankittipakul et al. 2012). More details, based on the sexual dimorphic characters of this genus, were not clearly revealed until Dankittipakul et al. published their work in 2012. Since then, species in the genus *Mallinella* started to be re-recognised by many surprising characters, such as chemosensitive hairs, proximal knob showing stridulating ridges, ordinary aculeate setae on femora I, chisel-shaped hairs on ventral part of tibia IV and the ventral spines in front of the spinnerets (Dankittipakul et al. 2012).

In the last three years, collections in Jiangxi Province in China led to the discovery of some new spider taxa, such as Agelenidae (Liu et al. 2021, Liu et al. 2020a), Corinnidae (Liu et al. 2022b), Phrurolithidae (Liu et al. 2020b, Liu et al. 2020c, Liu et al. 2021, Liu et al. 2022f), Salticidae (Liu et al. 2022a), Thomisidae (Liu et al. 2022c, Liu et al. 2022e, Li et al. 2022), Gnaphosidae (Liu et al. 2022d), Trachelidae (Zhang et al. 2022) and now Zodariidae (this study). These discoveries support the fact that this Province still has distributions of many unknown species because of the lack of systematic research and attention. When our team continued to study ground spiders from this Province, one undescribed and poorly-known species was found. The aims of the present paper are to provide detailed descriptions of this new species, based on specimens.

## Materials and methods

Specimens were examined using a SZ6100 stereomicroscope. Both male and female copulatory organs were dissected and examined in 80% ethanol using an Olympus CX43 compound microscope with a KUY NICE CCD camera. Epigynes were cleared with pancreatin solution (Álvarez-Padilla and Hormiga 2007). Specimens, including dissected male palps and epigynes, were preserved in 75% ethanol after examination. Types are deposited in the Animal Specimen Museum, College of Life Science, Jinggangshan University (ASM-JGSU).

The measurements were taken using a stereomicroscope (AxioVision SE64 Rel. 4.8.3) and are given in millimetres. The body lengths of all specimens exclude the chelicerae and spinnerets. Terminology of the male and female genitalia follows Dankittipakul et al. (2012).

Leg measurements are given as total length (femur, patella, tibia, metatarsus, tarsus). The abbreviations used in the figures and text are as follows: ALE − anterior lateral eye, AME − anterior median eye, Con – conductor, d − dorsal, EB – embolic base, Em − embolus, Fe − femur, ID − insemination duct, LB − lateral border, MA – median apophysis, MOA − median ocular area, p − prolateral, Pa − patella, PLE − posterior lateral eye, PME − posterior median eye, r − retrolateral, RTA − retrolateral tibial apophysis, Spe − spermathecae, Ti − tibia, v – ventral.

## Taxon treatments

### Mallinella shahu

Liu sp. nov.

C2D363F9-4881-5C55-991F-F368DF9F8FCB

AEB57C1F-6F88-4600-9DDA-281C52EB0C23

#### Materials

- **Type status:** Holotype. **Occurrence:** recordedBy: Liu Ke-Ke; individualCount: 1; sex: male; lifeStage: adult; occurrenceID: 274F2CC3-32F2-5C32-A2AF-64C4A00C6393; **Taxon:** scientificName: Mallinellashahu Liu, sp. n.; **Location:** country: China; stateProvince: Jiangxi; locality: Ji’an City, Suichuan County, Nanfengmian National Nature Reserve, Daijiabu Station, Shahu Village, Fengshuao; verbatimElevation: 1071 m; verbatimCoordinates: 26°16'1.33"N, 114°3'47.53"E; georeferenceProtocol: GPS; **Event:** samplingProtocol: sieving; eventDate: 28/06/2022
- **Type status:** Paratype. **Occurrence:** recordedBy: Liu Ke-Ke; individualCount: 2; sex: male; lifeStage: adult; occurrenceID: C9EC17EF-2286-5789-8809-B252BE1F4282; **Taxon:** scientificName: Mallinellashahu Liu, sp. n.; **Location:** country: China; stateProvince: Jiangxi; locality: Ji’an City, Suichuan County, Nanfengmian National Nature Reserve, Daijiabu Station, Shahu Village, Fengshuao; verbatimElevation: 1071 m; verbatimCoordinates: 26°16'1.33"N, 114°3'47.53"E; georeferenceProtocol: GPS; **Event:** samplingProtocol: sieving; eventDate: 06/28/2022
- **Type status:** Paratype. **Occurrence:** recordedBy: Liu Ke-Ke; individualCount: 1; sex: female; lifeStage: adult; occurrenceID: BAD600A6-1B24-5046-AC38-770954C8CE03; **Taxon:** scientificName: Mallinellashahu Liu, sp. n.; **Location:** country: China; stateProvince: Jiangxi; locality: Ji’an City, Suichuan County, Nanfengmian National Nature Reserve, Daijiabu Station, Shahu Village, Fengshuao; verbatimElevation: 1071 m; verbatimCoordinates: 26°16'1.33"N, 114°3'47.53"E; georeferenceProtocol: GPS; **Event:** samplingProtocol: sieving; eventDate: 06/28/2022
- **Type status:** Paratype. **Occurrence:** recordedBy: Liu Ke-Ke; individualCount: 1; sex: female; lifeStage: adult; occurrenceID: 1C8D4BC5-8CB6-5BDA-B3FC-746C9F4C5FA9; **Taxon:** scientificName: Mallinellashahu Liu, sp. n.; **Location:** country: China; stateProvince: Jiangxi; locality: Ji’an City, Suichuan County, Nanfengmian National Nature Reserve, Dafen Station, Gaoxing Village, Shiziao; verbatimElevation: 913 m; verbatimCoordinates: 26°20'28.85"N, 114°5'27.47"E; georeferenceProtocol: GPS; **Event:** samplingProtocol: sieving; eventDate: 06/27/2022
- **Type status:** Paratype. **Occurrence:** recordedBy: Liu Ke-Ke; individualCount: 1; sex: male; lifeStage: adult; occurrenceID: EB963720-873E-5D8C-B26B-A0BA01CAED6C; **Taxon:** scientificName: Mallinellashahu Liu, sp. n.; **Location:** country: China; stateProvince: Jiangxi; locality: Ji’an City, Suichuan County, Nanfengmian National Nature Reserve, DaijiabuStation, Shahu Village, Qingkenglong Hydropower Station; verbatimElevation: 1123 m; verbatimCoordinates: 26°15'26.42"N, 114°2'39.96"E; georeferenceProtocol: GPS; **Event:** samplingProtocol: sieving; eventDate: 06/28/2022
- **Type status:** Paratype. **Occurrence:** recordedBy: Liu Ke-Ke; individualCount: 1; sex: male; lifeStage: adult; occurrenceID: D1AE8B9F-4858-5386-9879-D9795C110395; **Taxon:** scientificName: Mallinellashahu Liu, sp. n.; **Location:** country: China; stateProvince: Jiangxi; locality: Ji’an City, Suichuan County, Nanfengmian National Nature Reserve, DaijiabuStation, Qianmo Village, Daaotou; verbatimElevation: 852 m; verbatimCoordinates: 26°17'17.10"N, 114°5'24.67"E; georeferenceProtocol: GPS; **Event:** samplingProtocol: sieving; eventDate: 06/28/2022
- **Type status:** Paratype. **Occurrence:** recordedBy: Liu Ke-Ke; individualCount: 1; sex: male; lifeStage: adult; occurrenceID: 3ED8AF9D-0331-5684-94EC-3B642839BC70; **Taxon:** scientificName: Mallinellashahu Liu, sp. n.; **Location:** country: China; stateProvince: Jiangxi; locality: i’an City, Suichuan County, Nanfengmian National Nature Reserve, DaijiabuStation, Qiaoshui Village; verbatimElevation: 542 m; verbatimCoordinates: 26°16'18.00"N, 114°5'40.99"E; georeferenceProtocol: GPS; **Event:** samplingProtocol: sieving; eventDate: 06/28/2022
- **Type status:** Paratype. **Occurrence:** recordedBy: Liu Ke-Ke; individualCount: 1; sex: male; lifeStage: adult; occurrenceID: DCA90A31-DF46-57AC-93D7-3D71076495F7; **Taxon:** scientificName: Mallinellashahu Liu, sp. n.; **Location:** country: China; stateProvince: Jiangxi; locality: Ji’an City, Anfu County, Taishan Township, Yangshimu, Yeniu waterfall; verbatimElevation: 541 m; verbatimCoordinates: 27°31'39.69"N, 114°14'37.18"E; georeferenceProtocol: GPS; **Event:** samplingProtocol: sieving; eventDate: 05/04/2021
- **Type status:** Paratype. **Occurrence:** recordedBy: Liu Ke-Ke; individualCount: 1; sex: female; lifeStage: adult; occurrenceID: F40FC25B-74A1-530D-B6F7-4A2EBFB51844; **Taxon:** scientificName: Mallinellashahu Liu, sp. n.; **Location:** country: China; stateProvince: Jiangxi; locality: Ji’an City, Anfu County, Taishan Township, Yangshimu, Yeniu waterfall; verbatimElevation: 542 m; verbatimCoordinates: 27°31'39.69"N, 114°14'37.18"E; georeferenceProtocol: GPS; **Event:** samplingProtocol: sieving; eventDate: 05/04/2021
- **Type status:** Paratype. **Occurrence:** recordedBy: Liu Ke-Ke; individualCount: 1; sex: female; lifeStage: adult; occurrenceID: 264C84C3-35D3-570A-9934-3E7B92AC212D; **Taxon:** scientificName: Mallinellashahu Liu, sp. n.; **Location:** country: China; stateProvince: Jiangxi; locality: Ji’an City, Taihe County, Ziyao Mountain;; verbatimElevation: 228 m; verbatimCoordinates: 26°43'5.30"N, 115°13'36.28"E; georeferenceProtocol: GPS; **Event:** samplingProtocol: sieving; eventDate: 10/28/2020
- **Type status:** Paratype. **Occurrence:** recordedBy: Liu Ke-Ke; individualCount: 2; sex: female; lifeStage: adult; occurrenceID: FA34B060-29E9-5877-B43E-11A55BF3319E; **Taxon:** scientificName: Mallinellashahu Liu, sp. n.; **Location:** country: China; stateProvince: Jiangxi; locality: Ji’an City, Taihe County, Ziyao Mountain; verbatimElevation: 338 m; verbatimCoordinates: 26°43'23.15"N, 115°13'31.70"E; georeferenceProtocol: GPS; **Event:** samplingProtocol: sieving; eventDate: 10/28/2020
- **Type status:** Paratype. **Occurrence:** recordedBy: Liu Ke-Ke; individualCount: 1; sex: female; lifeStage: adult; occurrenceID: C398B321-68C0-57CA-97CD-50457BB68BC9; **Taxon:** scientificName: Mallinellashahu Liu, sp. n.; **Location:** country: China; stateProvince: Jiangxi; locality: Ji’an City, Suichuan County, Bizhou Town, Baishuixian, Dakeng Formation; verbatimElevation: 329 m; verbatimCoordinates: 26°19'58.83"N, 114°44'00.01"E; georeferenceProtocol: GPS; **Event:** samplingProtocol: sieving; eventDate: 10/04/2019
- **Type status:** Paratype. **Occurrence:** recordedBy: Liu Ke-Ke; individualCount: 1; sex: female; lifeStage: adult; occurrenceID: EC481306-7D07-5ABB-A9E1-51215F797408; **Taxon:** scientificName: Mallinellashahu Liu, sp. n.; **Location:** country: China; stateProvince: Jiangxi; locality: Ji’an City, Jinggangshan City, Fuxi Village, Xiaoxi Forest Farm; verbatimElevation: 621 m; verbatimCoordinates: 26°29'32.24"N, 114°10'53.02"E; georeferenceProtocol: GPS; **Event:** samplingProtocol: sieving; eventDate: 11/14/2020
- **Type status:** Paratype. **Occurrence:** recordedBy: Liu Ke-Ke; individualCount: 1; sex: male; lifeStage: adult; occurrenceID: 56FD8CE0-EB31-5333-8E08-76808C7B3C7A; **Taxon:** scientificName: Mallinellashahu Liu, sp. n.; **Location:** country: China; stateProvince: Jiangxi; locality: Ji’an City, Jinggangshan City, Fuxi Village, Xiaoxi Forest Farm; verbatimElevation: 413 m; verbatimCoordinates: 26°28'22.92"N, 114°11'53.07"E; georeferenceProtocol: GPS; **Event:** samplingProtocol: sieving; eventDate: 11/14/2020
- **Type status:** Paratype. **Occurrence:** recordedBy: Liu Ke-Ke; individualCount: 2; sex: female; lifeStage: adult; occurrenceID: 0ABD40E8-A65E-570C-857E-EB6E613760C5; **Taxon:** scientificName: Mallinellashahu Liu, sp. n.; **Location:** country: China; stateProvince: Jiangxi; locality: Ji’an City, Jinggangshan City, Dalong Town, Yuantou Village; verbatimElevation: 550 m; verbatimCoordinates: 26°36'57.54"N, 114°5'21.08"E; georeferenceProtocol: GPS; **Event:** samplingProtocol: sieving; eventDate: 10/02/2019
- **Type status:** Paratype. **Occurrence:** recordedBy: Liu Ke-Ke; individualCount: 2; sex: female; lifeStage: adult; occurrenceID: D706C210-AEA2-5CD8-A771-742480C4582A; **Taxon:** scientificName: Mallinellashahu Liu, sp. n.; **Location:** country: China; stateProvince: Jiangxi; locality: Ji’an City, Taihe County, Yuanqian Town, Zhonglong Township, Ziyao Mountain; verbatimElevation: 394 m; verbatimCoordinates: 26°43'22.89"N, 115°13'31.78"E; georeferenceProtocol: GPS; **Event:** samplingProtocol: sieving; eventDate: 05/02/2021
- **Type status:** Paratype. **Occurrence:** recordedBy: Liu Ke-Ke; individualCount: 1; sex: male; lifeStage: adult; occurrenceID: 8C5E8ED7-D31A-511C-A914-EC047BACE518; **Taxon:** scientificName: Mallinellashahu Liu, sp. n.; **Location:** country: China; stateProvince: Jiangxi; locality: Ganzhou City, Dayu County, Nan'an Town, Shirenkeng, Daya Mountain Scenic Area; verbatimElevation: 459 m; verbatimCoordinates: 25°26'40.76"N, 114°21'00.21"E; georeferenceProtocol: GPS; **Event:** samplingProtocol: sieving; eventDate: 10/03/2020
- **Type status:** Paratype. **Occurrence:** recordedBy: Liu Ke-Ke; individualCount: 1; sex: female; lifeStage: adult; occurrenceID: 97C46CD4-0297-542E-9277-75B867EDC491; **Taxon:** scientificName: Mallinellashahu Liu, sp. n.; **Location:** country: China; stateProvince: Jiangxi; locality: Ji’an City, Taihe County, Laoyingpan Tunnel; verbatimElevation: 311 m; verbatimCoordinates: 26°34'32.34"N, 115°09'26.25"E; georeferenceProtocol: GPS; **Event:** samplingProtocol: sieving; eventDate: 11/05/2022
- **Type status:** Paratype. **Occurrence:** recordedBy: Liu Ke-Ke; individualCount: 1; sex: female; lifeStage: adult; occurrenceID: B6C25F65-F2EF-54C4-BC9C-7AF8D7020827; **Taxon:** scientificName: Mallinellashahu Liu, sp. n.; **Location:** country: China; stateProvince: Jiangxi; locality: Ganzhou City, Ningdu County, Cuiweifeng, Jinjing Cave; verbatimElevation: 346 m; verbatimCoordinates: 26°30'41.64"N, 115°59'19.02"E; georeferenceProtocol: GPS; **Event:** samplingProtocol: sieving; eventDate: 01/23/2021

#### Description

**Figure 1.**
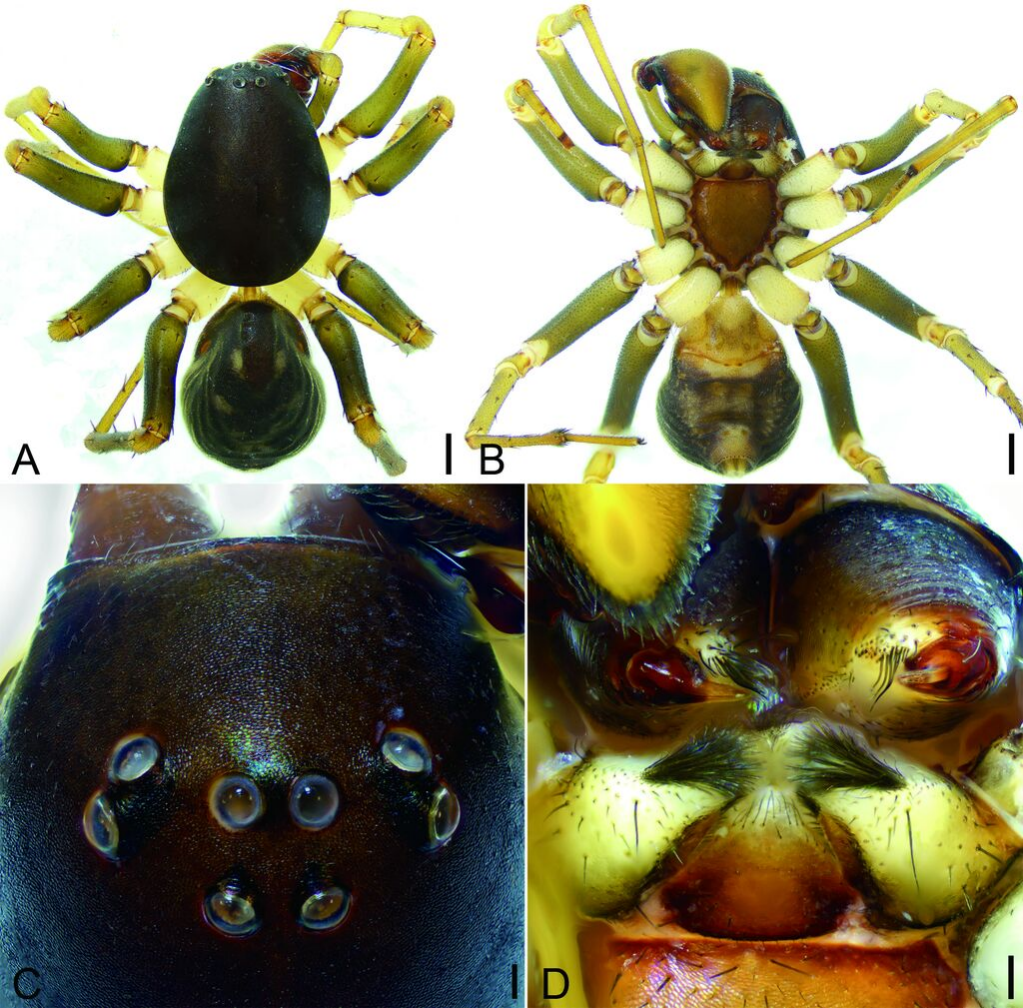
*Mallinellashahu* **sp. n.**, male holotype. **A** habitus, dorsal view; **B** same, ventral view; **C** eyes, dorsal view; **D** chelicerae and endites, ventral view. Scale bars: 0.5 mm (**A, B**); 0.1 mm (**C, D**).

**Male** (holotype) (Figs 1, 3A−D and Fig. 4A, B). Total length 5.35 mm.

Carapace (Fig. 1A, C) 2.91 mm long, 2.15 mm wide, anteriorly narrowed to half of its maximum width, with abundant short setae. Eye sizes and interdistances: AER and PER procurved in dorsal view; AME 0.13, ALE 0.14, PME 0.13, PLE 0.16, AME−AME 0.08, AME−ALE 0.19, PME−PME 0.12, PME−PLE 0.32, AME−PME 0.21, AME−PLE 0.28, ALE−ALE 0.64, PLE−PLE 0.91, ALE−PLE 0.06; MOA 0.43 long, front width 0.35, back width 0.39. Chelicerae without promarginal and retromarginal teeth. Endites triangular, longer than wide, anteriorly convergent, with dense brush hairs anterolaterally. Labium triangular, wider than long. Sternum strongly sclerotised, as long as wide, anteromedially with a wide notch, laterally with sclerotised serration-shaped margin, posterior end blunt. Legs (Fig. 1A, B): measurements: I 7.56 (1.97, 0.67, 1.78, 1.9, 1.24); II 5.95 (1.91, 0.71, 1.54, 1.79); III 7.04 (1.77, 0.76, 1.51, 1.92, 1.08); IV 8.61 (2.22, 0.84, 2.07, 2.5, 0.98); spination: I Fe: d3, p1; Ti: p1, v6; Mt: v7; II Fe: d3, p1; Pa: p1; Ti: p2, v7; Mt: v6; III Fe: d3, p3, r1; Pa: p1; Ti: d2, p2, r2, v6; Mt: d1, p3, r2, v8; IV: Fe: d4, p3, r1; Pa: p1; Ti: d1, p3, r3, v6; Mt: d1, p3, r4, v8. Pedicel (Fig. 1A, B) 0.15 mm long, cylindrical, sclerotised. Abdomen (Fig. 1A, B) 2.29 mm long, 1.83 mm wide, scutum covering less than half of dorsum; venter with a weakly sclerotised epigastric region anteriorly, two pairs of patches medially and a single row of ventral spines situated in front of the spinnerets.

Colouration (Fig. 1). Carapace black. Chelicerae brown to black. Endites yellow to brown. Labium and sternum reddish-brown. Legs yellow to light brown, femora darker than other leg segments. Pedicel dark yellow. Abdomen chestnut-brown, with four pairs of yellowish oval marks; venter yellow to brown, laterally with many small yellow patches.

Palp (Fig. 3A−D). Cymbium fold broad, strongly sclerotised, occupying slightly less than half cymbial length. Retrolateral tibial apophysis (RTA) digitiform, apex terminally bluntly pointed. Apical ridge strongly sclerotised on ventral side of palpal tibia. Median apophysis (MA) strongly curved forward on ventrolateral view. Conductor (Con) thick, tooth-like. Embolic base (EB) strongly curved laterally, embolus (Em) flattened, branching submedially; mesal ramus distinctly longer, relatively broad, terminally blunt, with two tooth-like apophyses subapically; lateral ramus relatively narrowed.

**Figure 2.**
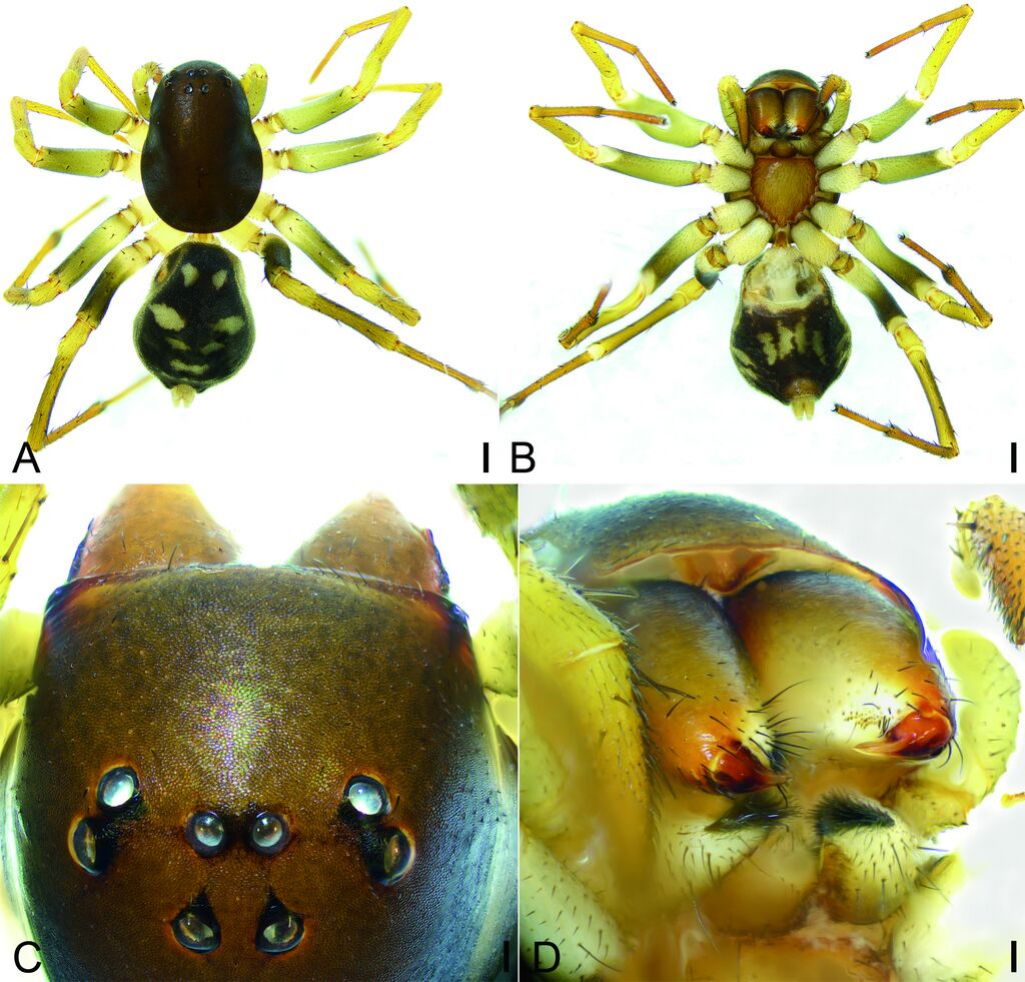
*Mallinellashahu* **sp. n.**, female paratype. **A** habitus, dorsal view; **B** same, ventral view; **C** eyes, dorsal view; **D** chelicerae, ventral view. Scale bars: 0.5 mm (**A, B**); 0.1 mm (**C, D**).

**Figure 3.**
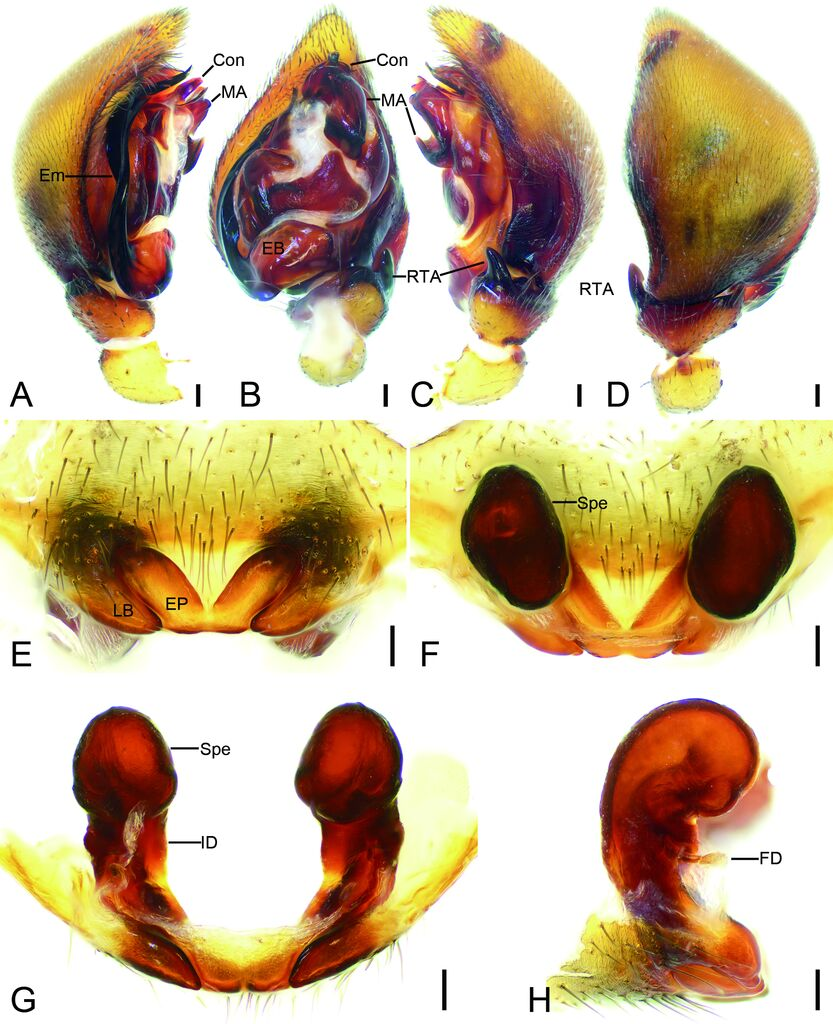
*Mallinellashahu* **sp. n.**, male palp of holotype and female epigyne of paratype. **A** palp, retrolateral view; **B** same, ventral view; **C** same, retrolateral view; **D** same, dorsal view; **E** epigyne, ventral view; **F** same, retrolateral view; **G** same, posterior view; **H** same, lateral view. Abbreviations: Con – conductor, EB – embolic base, Em − embolus, FD − fertilisation duct, ID − insemination duct, LB − lateral border, MA – median apophysis, RTA − retrolateral tibial apophysis, Spe − spermathecae. Scale bars: 0.1 mm.

**Figure 4.**
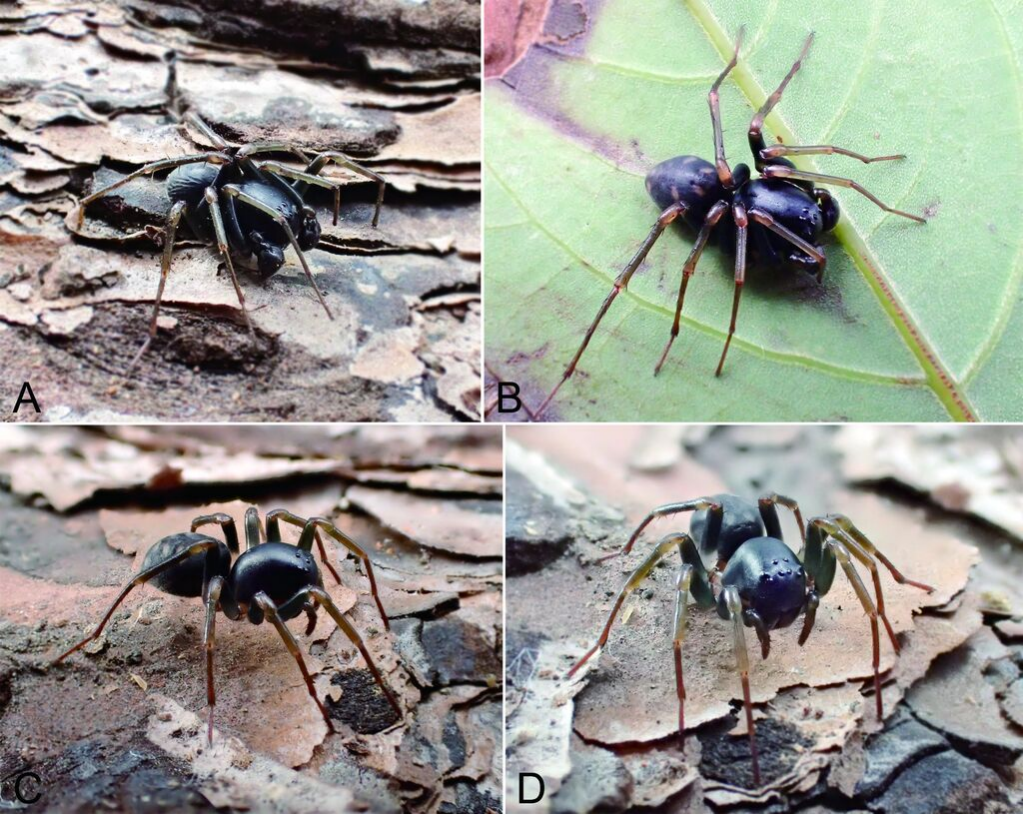
*Mallinellashahu* **sp. n.**, living specimen. **A & B** male; **C & D** female.

**Female** (Figs 2, 3E−H and Fig. 4C, D). As in male, except as noted. Total length 5.89 mm, carapace 3.05 mm long, 2.11 mm wide. Eye sizes and interdistances: AME 0.11, ALE 0.13, PME 0.12, PLE 0.14, AME−AME 0.1, AME−ALE 0.22, PME−PME 0.16, PME−PLE 0.33, AME−PME 0.25, AME−PLE 0.37, ALE−ALE 0.74, PLE−PLE 1, ALE−PLE 0.1. MOA 0.48 long, front width 0.31, back width 0.41. Leg measurements: I 6.77 (1.67, 0.72, 1.57, 1.5, 1.31); II 6.53 (1.82, 0.69, 1.33, 1.47, 1.22); III 6.88 (1.76, 0.78, 1.37, 1.83, 1.14); IV 8.77 (2.08, 0.8, 1.85, 2.56, 1.48); spination: I Fe: d3, p1; Ti: p1, v6; Mt: p1, v5; II Fe: d3, p1; Pa: p1; Ti: p2, v6; Mt: p2, v6; III Fe: d3, p3, r1; Pa: p1; Ti: d2, p2, r2, v5; Mt: d1, p3, r2, v7; IV: Fe: d4, p3, r1; Pa: p1; Ti: d2, p2, r2, v4; Mt: p5, r2, v5. Pedicel 0.09 mm long. Abdomen (Fig. 2A, B) 2.76 long, 2.15 wide, without scutum and ventral sclerite.

Colouration as in Fig. 2A, B. Abdomen paler than that of the male.

Epigyne (Fig. 3E−H). Epigynal plate (EP) broad V-shaped, median incision deep, almost reaching posterior margin. Lateral border (LB) along the lateral margin of epigynal plate heart-shaped. Insemination ducts (ID) pedicel-like, separated by their length, slightly parallel. Spermathecae (Spe) oval in dorsal view, ear-shaped in lateral view. Fertilisation ducts (FD) relative thin, as long as the width of insemination ducts.

#### Diagnosis

The male of this new species is similar to that of *Mallinellapseudokunmingensis* Yu & Zhang, 2019 (Yu and Zhang (2019): 4, figs. 1, 4, 6−18) in having a strong digitiform retrolateral tibial apophysis and the flattened embolus, but can be distinguished from it by the abdomen with four pairs of small yellowish marks (vs. four pairs of large yellowish marks) (cf. Fig. 1A; Yu and Zhang (2019): 4, fig. 1), the conductor with the blunt apex (vs. spine-like) (cf. Fig. 3A; Yu and Zhang 2019: 4, fig. 6), the thick median apophysis covering less than half of tegulum (vs. thin, more than or nearly half of tegulum) (cf. Fig. 3A−C; Yu and Zhang (2019) 2019: 4, fig. 7). The female of the new species resembles *M.pseudokunmingensis* (Yu and Zhang (2019): 4, figs. 2, 3, 5, 6, 19−23) in having the lateral border forming a heart-shaped structure and the oval spermathecae, but it can be easily distinguished by the anterior three pairs of separated small marks (vs. touching large marks) (cf. Fig. 2A; Yu and Zhang (2019): 4, fig. 2) and the W-shaped epigynal plate (vs. V-shaped) (cf. Fig. 3E; Yu and Zhang (2019): 4, figs. 19, 20) and the erect insemination duct (vs. slanting) (cf. Fig. 3G; Yu and Zhang (2019): 4, fig. 22).

#### Etymology

The species name is derived from the name of the type locality; noun in apposition.

#### Distribution

**Figure 5.**
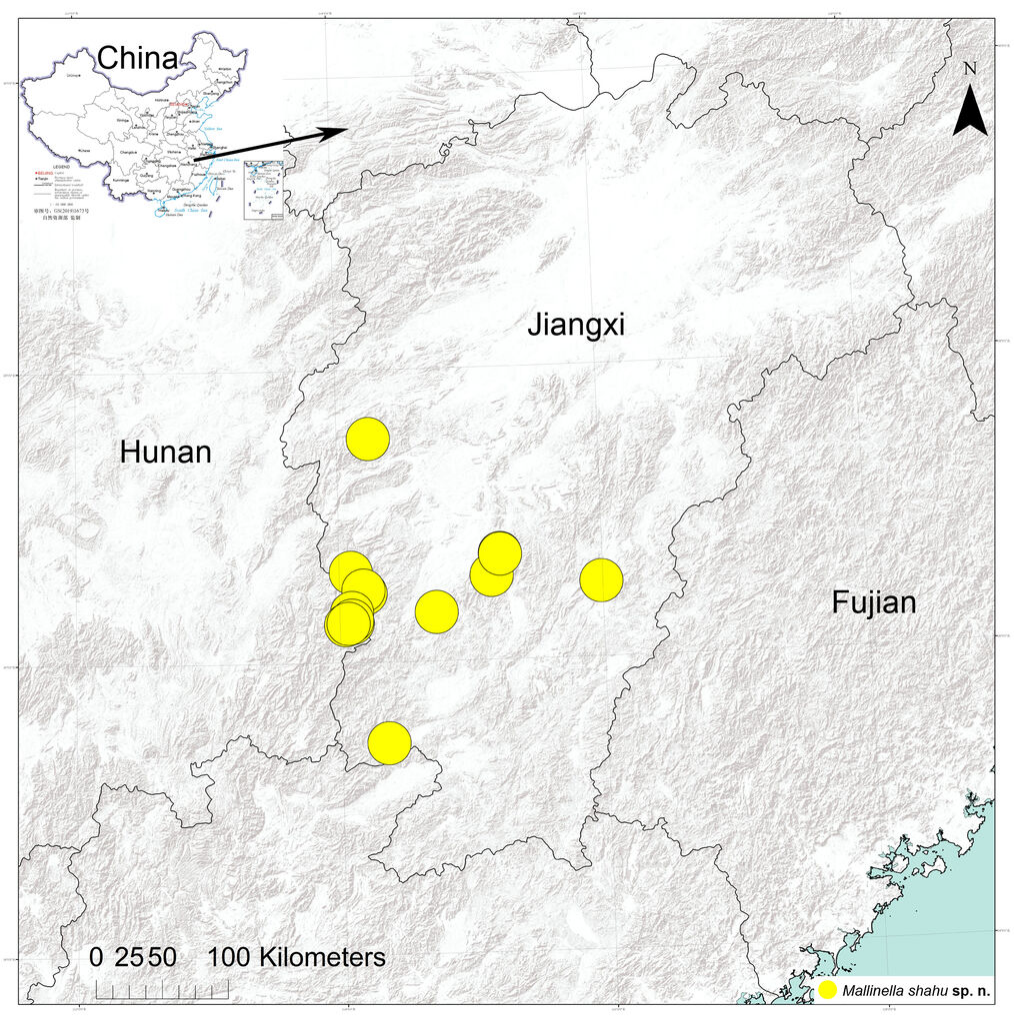
Records of *Mallinellashahu* sp. n., from Jiangxi Province, China.

Known from Ji’an and Ganzhou cities in Jiangxi Province, China (Fig. 5). It seems that this species has a wide distribution in this Province based on recent fieldwork.

#### Ecology

It was collected from leaf litter in areas of broad-leaved forests in hilly areas.

## Discussion

The genus *Mallinella* is divided into 22 species-groups by Dankittipakul et al. (2012), based on morphological characters; 202 species later, it becomes the most species-rich group in the family Zodariidae. According their localities, they are mainly distributed in Africa, the Indian subcontinent, Indo-Burma, Sundaland, Wallacea and Polynesia-Micronesia (Dankittipakul et al. 2012). Only 26 species (including the new species) have been reported from China (WSC 2023). The main reasons are that this group has not received much attention in China and that many *Mallinella* species are difficult to distinguish from their closely-related species, especially the females.

## Supplementary Material

XML Treatment for Mallinella shahu
